# Audit on Monitoring of National Early Warning Scores 2 (NEWS2) in Old Age Patients

**DOI:** 10.1192/bjo.2024.586

**Published:** 2024-08-01

**Authors:** Sadia Tabassum Javaid, Bethan Brace, Bethany Lee

**Affiliations:** North Staffs Combined Healthcare NHS Trust, Stoke-on-Trent, United Kingdom

## Abstract

**Aims:**

NEWS 2 is integral to post-admission physical health monitoring, guiding baseline establishment and observation frequency decisions. MDT discussions, involving medics or nurses per guidelines, ensure tailored care. Trust Standard Operating Procedure (SOP) and Physical Health policy, provides detailed procedures for assessment, recording, and actions. Adhering to NEWS 2 and SOP 1.62a, aligned with Trust standards, facilitates prompt escalation in case of patient deterioration, reinforcing our commitment to superior healthcare.

AIM

- To evaluate if NEWS2 monitoring is done as per set Trust standards/guidelines.
- To identify areas of improvement in the use of this observational tool.
- To improve the services and care of patients.

**Methods:**

We conducted a comprehensive review of each section of NEWS 2 charts for 39 patients admitted to Ward 6 and 7 at Harplands Hospital over a 3-week period. Patient stays varied from 21 to 67 days. No pregnancies were noted; all patients were aged between 59–96, with a near equal gender distribution. Utilizing SPSS, we conducted data analysis, comparing results against Trust-set standards.

**Results:**

Of the 39 charts, 37 were completed at admission, with notable issues: 9 lacked demographics, 13 had date/time missing. Weekly NEWS was predominant, but challenges included 6 missing signatures, 9 illegible entries, and 12 incomplete sections (4 without connecting observations). GCS completion issues were identified in two charts if CPVS score was more than 3. Escalation patterns varied: scores 1–4 were often routed to a Registered Nurse before medics, while scores >4 were mainly escalated directly to medics. Most charts were uploaded to electronic records, yet the electronic versions were frequently left unfilled.

**Conclusion:**

In conclusion, the implementation of NEWS charts at admission, consistent chart uploads to Lorenzo, and effective escalation practices underscore a commitment to patient monitoring. The detailed procedures, including demographics completion, trend identification, and weekly reviews, contribute to a comprehensive approach. The incorporation of printed patient information labels and targeted education sessions for ward teams further reinforces the emphasis on standardized and meticulous documentation practices, enhancing overall patient care and safety. Discussions with ward management will further support the ongoing success of these initiatives.

Above recommendation has been completed and Re-Audit in planned few months.

